# The smartHEALTH European Digital Innovation Hub experiences and challenges for accelerating the transformation of public and private organizations within the innovation ecosystem

**DOI:** 10.3389/fmed.2024.1503235

**Published:** 2024-11-29

**Authors:** Dimitrios G. Katehakis, Dimitrios Filippidis, Konstantinos Karamanis, Angelina Kouroubali, Anastasia Farmaki, Pantelis Natsiavas, Anastasia Krithara, Eleni G. Christodoulou, Marios Antonakakis, Dimitris Plexousakis

**Affiliations:** ^1^Center for eHealth Applications and Services, Institute of Computer Science, Foundation for Research and Technology – Hellas, Heraklion, Greece; ^2^Computational Biomedicine Laboratory, Institute of Computer Science, Foundation for Research and Technology – Hellas, Heraklion, Greece; ^3^eHealth Lab, Institute of Applied Biosciences, Centre for Research and Technology Hellas, Thessaloniki, Greece; ^4^Software and Knowledge Engineering Laboratory, Institute of Informatics and Telecommunications, National Center for Scientific Research “Demokritos”, Athens, Greece; ^5^Athena Research Center, Information Management Systems Institute, Athens, Greece; ^6^School of Electrical and Computer Engineering, Technical University of Crete, Chania, Greece; ^7^Department of Computer Science, University of Crete, Heraklion, Greece; ^8^Institute of Computer Science, Foundation for Research and Technology – Hellas, Heraklion, Greece

**Keywords:** digital transformation, electronic health, artificial intelligence, cybersecurity, high-performance computing, innovation ecosystems in health and care, place-based innovation, precision medicine

## Abstract

Digital innovation can significantly enhance public health services, environmental sustainability, and social welfare. To this end, the European Digital Innovation Hub (EDIH) initiative was funded by the European Commission and national governments aiming to facilitate the digital transformation on various domains (including health) via the setup of relevant ecosystems consisting of academic institutions, research centres, start-ups, small and medium-sized enterprises, larger companies, public organizations, technology transfer offices, innovation clusters, and financial institutions. The ongoing goal of the EDIHs initiative is to bridge the gap between high-tech research taking place in universities and research centres and its deployment in real-world conditions by fostering innovation ecosystems. In this context, the smartHEALTH EDIH started its operation in Greece in 2023, offering technical consultation services to companies and public sector organizations to accelerate digitalization in precision medicine and innovative e-health services by utilizing key technologies such as artificial intelligence, high-performance computing, cybersecurity, and others. During its first 20 months of operation, over 50 prospective recipients have applied for consulting services, mainly seeking “test-before-invest” services. This paper aims to provide insights regarding the smartHEALTH initiative, preliminary outcomes and lessons learned during this first period of operation. To this end, this paper outlines smartHEALTH’s approach to attracting recipients and providing expert guidance on utilizing state-of-the-art technologies for innovative services, product development, and process creation to accelerate digital transformation.

## 1 Introduction

The advent of the digital era is undeniable. The world is experiencing the Fourth Industrial Revolution, also known as Industry 4.0, which involves the integration of intelligent machines and systems, transforming production processes to enhance efficiency. In the healthcare sector specifically, emerging technical paradigms can provide huge prospects driven by digitalization, artificial intelligence (AI), and fifth generation (5G) telecommunications (1). Recognizing the importance of building a digital society, and as digital technologies offer new ways to citizens to learn, entertain, work, explore, and achieve personal ambitions, the European Union (EU) has reacted. The envisaged digital world should be founded on European values, ensuring that no one is left behind and that everyone enjoys freedom, protection, and fairness. To achieve this, the EU has set a goal for a Digital Decade, aiming to equip all citizens with the skills needed to use everyday technology (2). The EU’s Digital Decade provides a comprehensive framework that guides all actions related to digital space, ensuring that technology and innovation benefit everyone. In order to achieve the ambitious goals of the EU Digital Decade framework, the establishment of the Digital Compass was essential (3). The Digital Compass serves as a tool or guide that outlines specific goals and metrics for this transformation. It translates the ambitions of the Digital Decade into four key targets, known as the “four cardinal points,” for 2030. These targets focus on: (i) *Digital skills*: Ensuring that 80% of adults have basic digital skills and that Europe has 20 million ICT (Information and Communications Technology) specialists; (ii) *Secure and sustainable digital infrastructure*: Expanding high-performance connectivity, including 5G across Europe, and establishing secure and efficient digital infrastructure such as cloud computing and edge computing; (iii) *Digital transformation of businesses*: Helping 75% of EU companies adopt cloud computing, big data, and AI technologies. Also, increasing the number of tech-based start-ups and promoting innovation, especially among small and medium-sized enterprises (SMEs); and (iv) *Digital public services*: Ensuring that key public services are available online for citizens (e.g., citizens’ access to their electronic health records regardless of the country they are located to support cross-border healthcare services).

In line with the EU’s vision for digital transformation by 2030, the European Health and Digital Executive Agency (HaDEA) plays a pivotal role (4). HaDEA is responsible for implementing digital and health-related initiatives like EU4Health and Digital Europe, ensuring these program meet the objectives of the Digital Decade. The EU4Health program, with a budget of €5.3 billion for 2021–2027, aims to enhance healthcare systems through digital innovation, which is crucial to achieving the broader goals of the Digital Decade (5). The Digital Europe Program, with an overall budget of over €7.9 billion, provides funding for technological advancements that support these initiatives, including healthcare technologies, digital skills, and cybersecurity (6). These initiatives, among others, send a clear message that public health and the development of relevant digital health services is a priority for the EU.

This is also supported by initiatives like the European Health Data Space (EHDS) regulation, which will be a key pillar of a strong European Health Union. In spring 2024, the European Parliament and the Council reached a political agreement on the Commission’s proposal for the EHDS (7). The EHDS is expected to: (i) empower individuals to take control of their health data and facilitate the exchange of data for healthcare delivery across the EU (primary use of data); (ii) foster a genuine single market for electronic health record systems; and (iii) provide a consistent, trustworthy, and efficient system for reusing health data for research, innovation, policy-making, and regulatory activities (secondary use of data). The pressing need for the homogeneity and interoperability of European electronic health records is also highlighted by the Digital Decade 2024: eHealth Indicator Study (8). The main actions of the EHDS are organized in two pillars: (i) “Primary use” of healthcare data focusing on the ability of a European citizen to use his/her data across Europe, regardless of where these data are originally created/hosted to support cross-border healthcare services; and (ii) “Secondary use” of health data to support policy making and research for public health purposes.

Collaboration plays a key role in the success attained to date by networks of innovation ecosystems generated around entities known as European Digital Innovation Hubs (EDIHs), recently created following European Commission initiatives to boost the digitization of the European economic fabric (9). EDIHs are recognized as essential policy instruments designed to boost the digitalization of small and medium-sized enterprises (SMEs) and facilitate the transition to Industry 4.0 (10, 11). These hubs play a crucial role in fostering digital ecosystems across Europe, which consist of heterogeneous organizations spanning various economies, industries, and contexts. By offering a wide portfolio of supporting services, DIHs aim to enhance the digital capabilities of companies and drive innovation in regional economies (12). The Digital Europe Program supports the operation of European Digital Innovation Hubs (EDIHs) formed by strong collaboration between research and technology organizations (RTOs), universities, and representatives from the market such as innovative companies and business associations. The challenge of digital transformation is huge for companies in the EU. According to the Digital Decade targets set for 2030 the AI take-up is 11% of enterprises in 2023 against 75% of enterprises targeted for 2030 (13). EDIHs are expected to be a main actor boosting this process in the EU context (9, 14). Currently, there are 227 EDIHs of which 151 are funded directly by the Digital Europe Program. This Network of EDIHs is widely distributed across 85% of European regions, covering almost 90% of the EU’s working population. The services provided by EDIHs to SMEs and public sector organizations encompass a broad spectrum of technologies and sectors showcasing diversity in strategies and designs. The hubs demonstrate strong competencies in key technologies like AI, cybersecurity, and high-performance computing (HPC) (15).

As far as Greece’s population is concerned, this was 10,032,508 as of August 21, 2024 (16). The 2024 International Monetary Fund (IMF) figures show a gross domestic product (GDP) *per capita* of $23,966 (nominal) and $41,188 purchasing power parity (PPP) (17). Greece is recovering from a 10-year financial crisis and the COVID-19 pandemic, which heavily impacted its economy. Greece ranks 37th globally in the Global Innovation Index and is a “Moderate Innovator” (13). However, it ranks 25th out of 27 EU countries in the 2022 Digital Economy and Society Index (DESI) (18). Despite improvement, austerity measures still remain, and digital transformation is considered a key aspect which could facilitate businesses to be competitive, especially in the post-pandemic era.

The Digital Decade 2024 report highlights Greece’s efforts to improve its digitalization, especially in e-health, which has surpassed national targets for 2023 (19). The country’s digital transformation roadmap, supported by EU funds, focuses on enhancing healthcare and cross-border e-health services, including electronic medical records. In the same report, it is mentioned that the national targets set for e-health Greece’s align with the EU’s 2030 goals, surpassing its 2023 forecast with a score of 73.8 and 21.6% annual growth. The health sector’s digital transformation is a priority in Greece’s national strategy, supported by EU funds through the Recovery and Resilience Facility (RRF) (20). Of the €5.23 billion allocated to support the domain of health services, €394.8 million have been allocated to support e-health infrastructures.

Along these lines, the interconnection between research and academic institutions and industry/public sector is highlighted as a crucial factor in terms of providing know-how gained via international collaborations, research projects etc. to the industry in order to capitalize in real-world services and/or products. In this context, smartHEALTH brings together expertise in the areas of AI, cybersecurity, and HPC, to facilitate digital transformation and foster innovation in digital health in Greece. This article focuses on the smartHEALTH test-before-invest services (TBI) and outlines the relevant private/public organizations participating in the project as “service recipients,” i.e., organizations who receive consulting services highlighting the areas of activity and the preliminary results from its initial 20 months of operation. It also discusses the challenges it confronted, lessons learned so far in terms of applying novel technical paradigms in real-world conditions, and outlines next steps.

## 2 Materials and methods

The initiative of EDIHs aims to bridge the gap between research and market deployment by fostering ecosystems where innovation can thrive. The smartHEALTH setup and operation framework selected for community engagement, as well as the methodology selected for the brief presentation of research outcomes are detailed in the following subsections.

### 2.1 The smartHEALTH approach

As already mentioned, the Network of the EDIHs operates across Europe with the support of the European Commission, bringing together relevant national initiatives, SMEs, and public sector organizations (PSOs) to make the EU’s Digital Decade 2030 targets a reality. The EDIH Network is a community of tech experts dedicated to guiding Europe’s businesses and public sector organizations on their path to digital transformation. EDIHs serve as one-stop shops throughout all EU regions, equipping companies with the essential digital tools to improve their competitiveness, upgrade their infrastructure, and boost their overall success (21). The mission of EDIHs is three-fold:

- Advance digital transformation across the EU by bringing cutting-edge tech (AI, Cloud, Big Data) to 75% of European companies.
- Ensure that 90% of companies have a basic level of digital know-how.
- Create new value chains within Europe.

**Figure 1:**
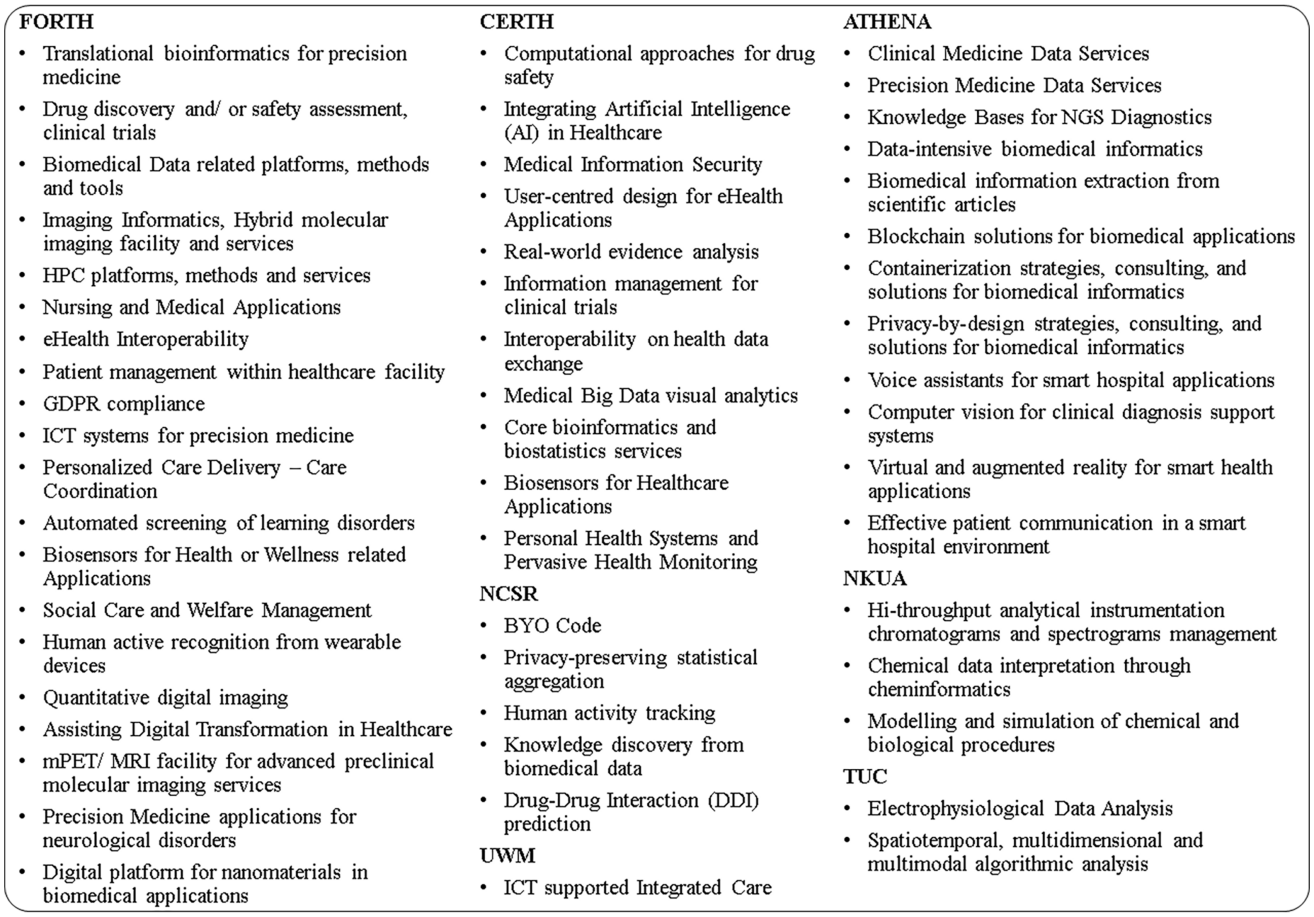
TBI expertise areas for services offered by smartHEALTH grouped by each of the service provider organizations.

SmartHEALTH is designed as a “one-stop-shop” where SMEs, start-ups, mid-caps, and the public sector can get help to improve their processes, products and services by means of digital technology. The hub is co-funded by the EC and the Greek State and brings together some of the main Research and Innovation leaders of the Greek ecosystem in the field of digital and smart health to facilitate the digital transformation of the private and public sector (22). Prominent actors in the fields of research and innovation (Foundation for Research and Technology - Hellas (FORTH), Centre for Research and Technology Hellas (CERTH), National Centre for Scientific Research Demokritos (NCSRD), ATHENA Research Center), in tertiary education (National and Kapodistrian University of Athens (NKUA), University of Crete (UOC), Technical University of Crete (TUC), University of West Macedonia (UWM)), an experienced ICT -integration company (UNISYSTEMS) and a business support organization (SEVPDE) have joined forces to offer a large variety of specialized services in 54 main areas of expertise and 3 main technological fields, namely AI, Cybersecurity and HPC. The list of the main areas of expertise which can support smartHEALTH TBI services is shown in Figure 1.

The list of TBI expertise areas for services depicted in Figure 1 is not exhaustive and portrays the main expertise available in the knowledge intensive organizations that form the core of the hub. The scheme develops state of the art methodologies and strategies based on years-long research on the needs of EU and worldwide representative ecosystem stakeholders. The expertise utilized for each recipient is based on their real needs and may differ.

In line with the overall implementation strategy of the EDIH Network, smartHEALTH provides a portfolio of four distinct types of services. More specifically:

- *TBI services* to SMEs and the public sector to assist in their digital transformation and the support of innovation, through added-value technical consultation and use of state-of-the-art infrastructure. TBI services include (i) Awareness raising on available digital technologies, and implementation of a “Digital Maturity Assessment” (DMA) using a structured tool provided by the EC for measuring the digital maturity of the client, (ii) the elaboration of a specific plan of action for further services based on a “Visioning for digital transformation” exercise, the DMA results and a Strengths-Weaknesses-Opportunities-Threats (SWOT) and market trends analyses, (iii) “Demonstration and proof-of-concept” in which SMEs and public sector entities demonstrate in a lab the idea behind a new product, service or process and smartHEALTH experts assess the validity and feasibility of the new concept providing additional support, (iv) “Fostering integration” in which smartHEALTH experts support clients to integrate digital technologies and skills in their processes and products, (v) “Testing, experimentation and prototyping” through which clients have the opportunity to pilot test innovative technologies and products using available infrastructure with the guidance of skilled smartHEALTH personnel, and (vi) “Flagship” services of increased importance and added-value in the specific areas of precision medicine, cancer, and the digital transformation of the public sector.
- *Skills and training services* to SMEs and the public sector to assist in their personnel’s digital skills development. These services include the organization of seminars and workshops, tailor-made specialized training courses, hackathons, bootcamps and other competitions.
- *Access to public and private financing services* to SMEs and the public sector, to assist them in securing financial resources for the implementation of digital transformation activities. These services include 1–1 coaching and mentoring on available public and private funding opportunities, as well as advanced consultation on preparing funding proposals, pitch-decks, business plans, etc. At the same time, smartHEALTH has set up an extensive support network of financial institutions, such as banks, private funding bodies, venture capitals, angel seeds, etc., which is being used for connection with recipients seeking funding.
- *Ecosystem development and networking* services to support, facilitate, and animate the digital health ecosystem in Greece and empower all relevant actors. smartHEALTH organizes partnering events and participates in common activities with the EEN, clusters, and other key stakeholders with the aim to foster synergies and promote collaborative projects with partners from the quadruple helix.

In this paper, results for the TBI type of services will be presented and analysed since they lie at the core of the smartHEALTH service portfolio and provide its unique selling proposition.

### 2.2 Framework for community engagement and its application

The engagement of interested organizations from the Greek ecosystem is being performed through different channels such as: (i) contact to or from smartHEALTH experts, (ii) the submission of an online application, (iii) online inquiry through the contact form on the smartHEALTH website.

After the initial contact is made, a clear workflow for the provision of services ensues with a structured process of operation. Service provision is divided into 4 distinct but interrelated stages:

- *Preparatory stage*: This entails introductory meeting(s), online submission of application, evaluation of application by the smartHEALTH Management Board, preparation and signing of first Cooperation Agreement
- *Stage 1*: This entails the successful provision of Phase 1 TBI services (awareness raising, digital maturity assessment, and visioning), the delivery of a technical report to the client, and a signed certificate of delivery and acceptance by them.
- *Stage 2*: This entails preparation and signing of additional Cooperation Agreements and the provision of Phase 2 TBI services (demonstration/proof of concept, fostering integration, testing/prototyping/experimentation, flagship). In addition, delivery of a detailed technical report to the client.
- *Stage 3*: This entails the impact analysis of provided services, as well as customer satisfaction, including quality control and assessment, through a signed certificate of delivery and acceptance by the client, as well as an optional automated rating scheme.

**Figure 2:**
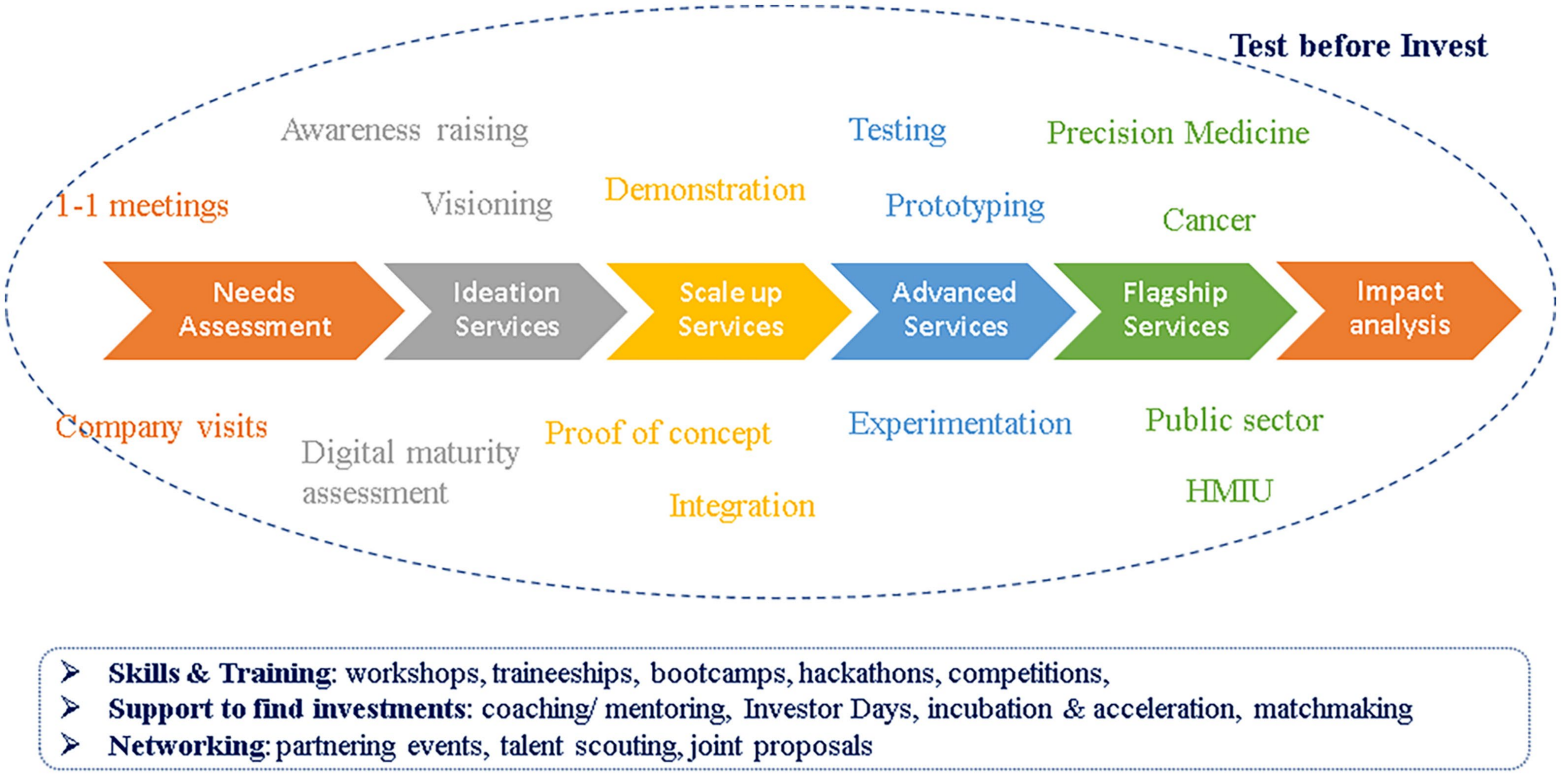
Client journey. The steps from the first meeting with the potential client to the analysis of the impact of the provided service. Scale up, advanced and flagship services are provided only as needed.

The overall client journey is depicted in Figure 2.

In parallel to the provided TBI service, the entire set of other EDIH services activities are run based on recipients’ needs, offering the opportunity for collective training services on areas of common interest as well as networking/matchmaking between the community stakeholders. This way smartHEALTH provides to service recipients both collectively as well as individualized access to specialized knowledge and expertise, together with support to find investments.

### 2.3 Data collection process

The authors gathered data from the Client Relationship Manager (CRM) system developed by smartHEALTH, which is used by the private companies and public organizations acting as TBI service recipients. The CRM system is used by these organizations to submit their application, and smartHEALTH consortium to manage and report on the provided services. The organizations which can receive services from the smartHEALTH (inclusion criteria) are public and private organizations that have (i) initiated the application process at the online dedicated platform; (ii) submitted a formal application, accompanied by all the required formal documents; (iii) been positively evaluated (for financial viability and satisfying the pre-conditions for inclusion in the program); (iv) selected at least one TBI service; and (v) signed a cooperation agreement with smartHEALTH. There was no limit placed on the type of service or technology involved.

The data screening procedure involved two rounds. The following data elements were extracted from the EDIH database for all open applications: entity applied to EDIH, category of service requested, type of legal entity, staff size, region based on the nomenclature of territorial units for statistics 2 (NUTS2) classification (23), phase (for contracted entities only), type of services requested (for contracted entities only), digital technologies involved (for contracted entities only), and short description of the requested services. Authors screened the data, in order to eliminate possible errors or bias in the selection process. The names of all clients were removed and all relevant data retrieved were assessed for their eligibility according to the inclusion and exclusion criteria. Data was processed, codified, and analysed in order to provide an outline of the EDIH recipients’ profiles, as well as the existing demand.

In the sections below, current progress and lessons learnt from the provision of TBI will be presented, while significant barriers toward the EDIH adoption and integration into the wider innovation ecosystem will be identified.

## 3 Results

In its first 20 months of operation, smartHEALTH achieved significant milestones. The hub provided added-value TBI services to a diverse range of organizations, including SMEs, civil society organizations, and public sector organizations, such as hospitals. Additionally, through the organization of seminars and workshops, smartHEALTH created valuable opportunities for participants to deepen their knowledge in the field of digital health and engage with the broader research ecosystem. In parallel, smartHEALTH organized and participated in numerous networking events in order to foster cooperation and create synergies between different actors of the ecosystem.

**Figure 3:**
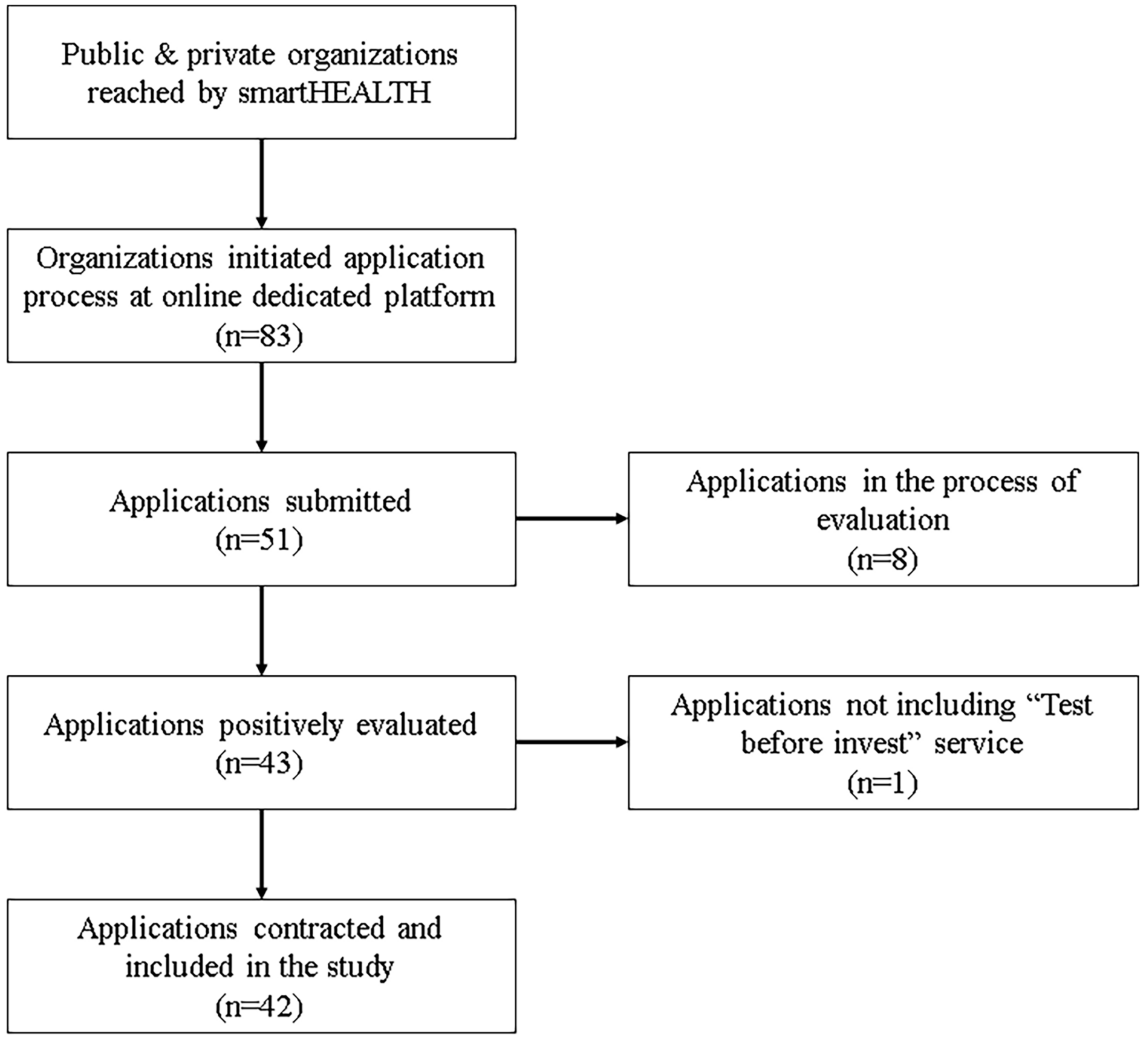
Flow diagram for study inclusion. Number of applications and TBI contracts in each phase.

As of 23 August, 2024, 83 public and private organizations had initiated the process of submitting a formal application for support services by smartHEALTH experts. Data from 51 applicants had been collected from the smartHEALTH CRM platform, following their formal application submission. Eight of them were in the process of evaluation by the smartHEALTH Management Board (MB). Forty-three had submitted an application which had been positively evaluated. Forty-two included a request for the delivery of a TBI and were incorporated into the study. Those that did not include a TBI service request, as well as those that for any reason did not get approval by the Management Board of smartHEALTH were excluded. This process is depicted in Figure 3. The data set for the performed analysis and outcomes are detailed in the Supplementary Table S1.

A preliminary analysis of the filtered data has produced the following results:

- With regards to differentiation between private and public organizations which have applied for smartHEALTH services, there is a significantly larger proportion of private organizations that have applied (86–14%), and consequently that have received services (38 private and four public organizations, out of 42 total). This gap could be attributed to public sector bureaucratic constraints, as well as non-prioritization of the adoption of digital technologies and lack of awareness of their benefits.
- Concerning geographical coverage, there is a much stronger interest from organizations based in Attiki, Central Macedonia, and Crete (45 out of the 51 applicants and 38 out of 42 contracted entities, were from these three regions). Interest in other parts of Greece is much lower. This can be attributed to the concentration of the majority of project partners/service providers in these three regions, but more importantly to a relevant gap in digital maturity level as well as financing and business opportunities that exists between metropolitan centres (Athens, Thessaloniki, Heraklion) and the periphery.
- The size of applicant organizations also provides interesting results. Micro and small size organizations are responsible for 82% of the submitted applications, while only 18% are medium and large size entities. When looking at contracted organizations, the difference is even larger with 86% being micro or small size, while 14% being medium or large size. The reasons vary, from a stronger innovative capacity and interest of small companies (such as start-ups) that want to take the next step in their development, to a relative lack of interest in smartHEALTH services from larger organizations that have other means to promote their digital transformation.
- The types of advanced TBI services that were requested do not provide any significant realizations. Almost half of the requested services had to do with demonstration and proof of concept (20 out of 45), while 12 requests involved testing/prototyping/experimentation, and 11 involved fostering integration. Only two cases qualified as flagship services.
- Concerning technologies used, there is a strong preference for Software Architectures, AI and Decision support, and Internet services and applications. This is again not surprising given the fact that software development for internet services and apps is the core business in the ICT sector, while AI has been the focal technological sector of the last few years. Although not surprising, this preference for established technologies shows the relative conservativism that permeates business culture in Greece.

**Figure 4:**
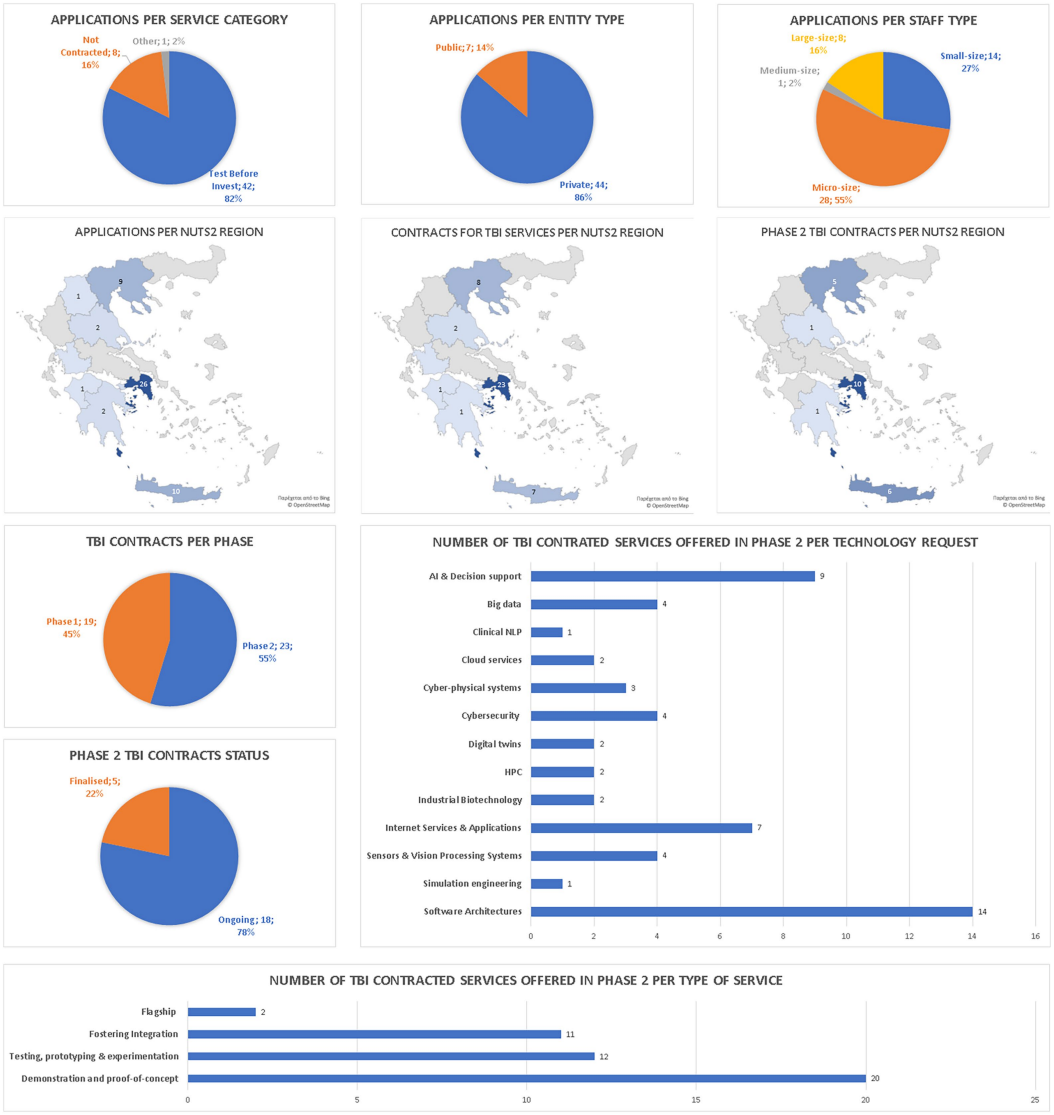
The EDIH services offered be type/size/region of recipient, as well as type of service requested, and technology used. Top row: Breakdown of TBI services per entity type and per client company size. Middle row: Geographical spread of (left) the successful TBI service applications on CRM, containing the contracted and the ones that are about to be contracted, (centre) the contracted (phase 1 and 2) TBI services and (right) the TBI services that have proceeded to phase 2. The bottom left pie charts provide a more detailed visualization of the stage of TBI contracts under phase 1 and 2. The bottom right and bottom down bar charts show a breakdown of TBI services per technology request and per type of phase 2 service.

A schematic depiction of EDIH services offered by type/size/region of recipient, as well as type of service requested, and technology used, can be found in Figure 4. This is central to the article objectives since it illustrates preliminary smartHEALTH analysis outcomes, discussed in the previous paragraph.

An indicative example of the services provided by smartHEALTH is the published success story of HERADO SA (24). The Greek company offers a disruptive solution for radiation protection, a patented active dosimeter. The dosimeters connect to a dedicated monitoring and reporting platform, through Internet of Things (IoT). The platform is developed from a third-party, with no knowledge of the health requirements concerning privacy, interoperability and cybersecurity. The company addressed smartHEALTH to check if the platform was complying with the needed set of standards for Health IT software. Another concern for the company was how to expand the platform for responding to an expected scaling-up of users and an addition of future services. smartHEALTH experts reviewed the platform in a series of interviews with the company. Then, it provided a detailed report in which they listed what the platform should have and specific proposals for future developments. This case is similar to many other in which Greek SMEs address smartHEALTH for receiving a proof of concept and advice for their software. There is no other body in Greece providing such services.

## 4 Discussion

Transitioning into its second year of operation, significant insights were gained for the Greek eHealth ecosystem, highlighting a strong desire for digital integration and enhancement of services and products through innovative digital approaches. The free provision of consulting services by smartHEALTH, facilitated by support from the EU and the Greek state, emerged as a vital facilitator for the EDIH’s operations. However, challenges regarding the workload of service provision were identified as significant barriers to overcome during this period, as well as administrative issues relevant to guaranteeing national funding. Large capital companies were hard to reach and collaborate with, since all large size organizations depicted in Figure 4 are PSOs. In contrast small companies, start-ups etc. are open to collaboration. Despite the fact that there is a huge investment in eHealth in Greece through RRF, smartHEALTH did not manage to have contractors incorporated as recipients of TBI services. This lack of involvement for this type of companies could imply lack of capacity as this is a very busy period for them or lack of interest for the adoption of innovative technologies, lack of collaboration mentality, or fear for exposing potential commercial secrets.

Some important findings/lessons learnt from the 20-month period have to do with the fact that since the focus of TBI services rely on the fulfillment of the needs for digitalization of recipients with different and in many cases not fully crystalized agendas, the ability to adapt to changing customer needs is crucial. This requires a business mentality which is not evident to all researchers or academia professionals. Nevertheless, it is exactly those who are closely situated to knowledge production (such as RTOs and academia) that can provide the confidence and build long term relationships and partnership with the clients that seek continuous improvement. In addition, researchers need to have clear incentives and understanding that through the EDIH they do not conduct research, but rather assist the ecosystem innovate and set the ground for stronger partnerships. Preliminary findings indicate also that red tape remains still an issue as well as the identified difficulty in attracting PSOs.

The first-year achievements of smartHEALTH, in the direction of supporting businesses and the public sector on their path to digital transformation, are quite encouraging for the coming years’ operation. To this end, multiple ways to achieve sustainability are currently being investigated. The operation of smartHEALTH has unveiled several key advantages, demonstrating the value of uniting efforts for shared goals. First, by working together, researchers and the service recipients reached into innovation areas that had a wider impact in the digital transformation of each organization. The well-structured application process that defined the purpose of each collaboration, identifying the appropriate multidisciplinary team to address it and aligning it with the performance indicators of the hub became seamless. Within the national context, smartHEALTH simplified activity planning, given the shared work culture and emphasized research and public-private partnerships. Any emerging issues were addressed using the combined operational and communication cultures the organizations involved, fostering innovation and problem-solving. The allocation of resources was based on principles of efficiency and effectiveness, achieved through meaningful dialog between the EDIH service recipients and the hub organizations. The collaboration has helped into creating a deep understanding of each organization’s characteristics, as well as prior experiences. Particularly crucial was the involvement of researchers who had targeted expertise facilitating smoother interactions. An initial agreement, including a tentative timeline for implementation and a clear definition of roles, was essential to the collaboration’s success. Furthermore, the strategic promotion of the partnership helped reinforce its value.

This collaboration is now recognized as a best practice model for other hubs with complementary goals across the country. However, one of the main challenges encountered was the diversity of perspectives among the various EDIHs, each serving different sectors and regions with unique priorities. While smartHEALTH focuses on digital health, other hubs have focused on agriculture, energy, the environment, and the public sector, leading to differing views on the ecosystem’s most pressing challenges and how to address them. Despite these differences, these divergences were ultimately seen as opportunities for growth. Through collaborative actions, the hubs were able to align on shared goals, leverage their complementary strengths, and contribute to a more holistic national digital transformation strategy. Though sectors differ, cross-cutting challenges such as cybersecurity, data management, and workforce digital upskilling emerged as shared areas of concern. AI, data analytics, and IoT, are at the core of all EDIHS and are relevant across sectors. Cross-pollination of ideas contribute to the expansion of the innovation ecosystem. The collaboration fostered a more unified approach to digital transformation in Greece by recognizing that each EDIH’s unique focus could contribute to an overall national innovation strategy.

## 5 Limitations

The conclusions are subject to several limitations. First, all findings are based on a small dataset, restricted to the Greek healthcare ecosystem. Additionally, the approach followed by the authors might have influenced the results, as the concept of EDIHs is relatively new and smartHEALTH is still in its early stages. Furthermore, the majority of services offered so far have not been fully completed. Information about the overall service impact monitoring for all beneficiaries will require more time, along with plans to engage/support the beneficiaries on the long term. The EDIH received very few spontaneous applications; most were generated through discussions and engagements at scientific events and forums. This approach may distort the understanding of the practical needs of the Greek ecosystem.

## Data Availability

The original contributions presented in the study are included in the article/Supplementary material, further inquiries can be directed to the corresponding author.
