# Supplementary material for: The smartHEALTH European Digital Innovation Hub experiences and challenges for accelerating the transformation of public and private organizations within the innovation ecosystem
Source: Front Med (Lausanne). 2024 Nov 29;11:1503235. doi: 10.3389/fmed.2024.1503235 (PMC11638864; doi:10.3389/fmed.2024.1503235)
Supplement: Supplementary file 1 [file Table_1.DOCX]

| no | type | size | region | phase | type of service | technology used | short description | status |
| --- | --- | --- | --- | --- | --- | --- | --- | --- |
| 1 | private | small-size | Dytiki Makedonia | - | - | - | - | - |
| 2 | private | small-size | Kentriki Makedonia | - | - | - | - | - |
| 3 | private | micro-size | Attica | 1 | - | - | - | - |
| 4 | private | small-size | Attica | 1 | - | - | - | - |
| 5 | private | small-size | Attica | 1 | - | - | - | - |
| 6 | private | small-size | Attica | 1 | - | - | - | - |
| 7 | private | micro-size | Kriti | 1 | - | - | - | - |
| 8 | private | medium-size | Attica | 2 | D | SA | The company provides occupational doctor services to companies and develops a platform to digitalize all the procedures needed for this provision, such as preventive periodic examination of workers, establishment of medical records of the personnel, health assessment of work capacity, preparation of vaccination programs, cooperation for the formation of a list of procedures for the safe execution of works, training and information of workers etc. EDIH experts consult the company for the non-operational characteristics of the platform, especially security, interoperability and privacy. Experts also look at the software architecture and provide recommendations. | ongoing |
| 9 | private | small-size | Kentriki Makedonia | 1 | - | - | - | - |
| 10 | private | micro-size | Kriti | 2 | D, T | AI&DS, CPS, ISA | The company develops an eye-tracking tool, as a diagnostic tool for learning disabilities and dyslexia. The company will present its solution and smartHEALTH experts assess the product and provide technical consultation regarding the processing method the tool uses. The company receives a report concerning the findings, especially related to the accuracy of the tool. The company works together with smartHEALTH experts to jointly develop machine learning models (random forests, extreme gradient boosting) and applied statistics (supervised learning models for high dimensional data, including penalized logistic regressions). These models are integrated into the system to demonstrate new functionalities. The company experiments with them and smartHEALTH experts help them record and analyze the results of the experimentation. | ongoing |
| 11 | private | micro-size | Attica | 1 | - | - | - | - |
| 12 | private | micro-size | Attica | 1 | - | - | - | - |
| 13 | private | micro-size | Peloponnisos | - | - | - | - | - |
| 14 | private | micro-size | Attica | 2 | D, I | SA | The company presents the recovery diary it has developed, currently in paper form, and gets feedback from smartHEALTH experts about its digitization. Its non-functional characteristics are discussed. These include interoperability with other systems, security, privacy, and conformance to regulations. Experts from smartHEALTH determine the specifications the recovery diary software should have. In addition to specs, smartHEALTH experts work with company representatives to define and develop a design prototype (mockup) of how the company would like the application to be developed to look like. The specifications, along with the prototype, can thus be outsourced to a third party, who will then be able to undertake the implementation of the application. | ongoing |
| 15 | private | micro-size | Kentriki Makedonia | 2 | D, T | SA, CPS, SVPS | The company designs, produces and sells simple rehabilitation equipment. The company is now ready to take the leap and design new rehabilitation equipment that will allow the transfer of medical data from the patient to the doctor. This effort is supported by smartHEALTH which assists the company across two dimensions: (i) to determine the software requirements concerning non-functional characteristics (privacy, safety, interoperability, architecture), and (ii) to help the company perform testing, experimentation and prototyping with sensors that will monitor a series of vital signals to be transferred to the doctor. | ongoing |
| 16 | private | small-size | Attica | 2 | D, T | SA, CPS, CYB | The company develops a software tool for suggesting the proper radiation dose for children that need to have an MRI. Experts from smartHEALTH examine the software, concerning its non-functional characteristics. These include the issues of security, privacy, interoperability, standards and architecture. A report is provided with the findings of the assessment. The company also needs to test and further develop their MRI system. In that direction smartHEALTH experts evaluate the system, based on technical criteria and efficiency with appropriate models. The experts work together with the company to design and experiment new features that could be added to optimize the system. The experts advise the company on data analysis issues, after the tests they carry out and propose improvements at the software level of both system control and data analysis. | ongoing |
| 17 | private | micro-size | Thessalia | 1 | - | - | - | - |
| 18 | private | micro-size | Attica | 2 | D, I | SA, HPC, BD | The company offers innovative services in bioinformatics, next generation sequencing and biomedical data analysis. The consulting services provided by smartHEALTH focuses on ensuring the quality control of the company's systems. The recommendations address interoperability and adaptation to the local environment, security and protection of personal data, and consultancy on standards for storing, sharing, and analyzing genomic data in a cloud environment. | ongoing |
| 19 | private | micro-size | Attica | 1 | - | - | - | - |
| 20 | private | small-size | Kriti | 2 | D, I | SA, ISA | The company wants to develop a new CRM, tailored to specific needs. This CRM requires to have an EHR section for which the company needs to know exactly how to develop it. Experts from smartHEALTH check the approach of the company and give advice concerning several issues of privacy, safety, interoperability and standards. The company also needs to design the CRM with suitable operations needed. smartHEALTH experts provide new unmet operations and help the company. In the end of the collaboration, the client has the design of the CRM system needed. | ongoing |
| 21 | private | micro-size | Attica | - | - | - | - | - |
| 22 | private | micro-size | Attica | 2 | D, I | SA, BD | The company is an SME with expertise that spans across a variety of statistics and computer science domains. EDIH experts provide the company with a report containing recommendations to address their needs in organizing bioinformatics workflows, efficiently managing of the VCF files, integrating and standardizing information sources for clinical genome analysis and also consulting guidance to scale up the company. | ongoing |
| 23 | private | micro-size | Attica | - | - | - | - | - |
| 24 | private | micro-size | Kriti | 2 | F | IBT | The company works with biological samples analysis. Analysis of blood, urine, hair and tissue samples can provide valuable information about exposure to a wide variety of toxic substances. smartHEALTH provides support for the pre-clinical testing of the biodistribution of cannabinoids, with a simple and innovative method of biological sample analysis. The method is promising to provide a simple, yet effective tool for tracing cannabinoids. | ongoing |
| 25 | private | small-size | Attica | 1 | - | - | - | - |
| 26 | private | micro-size | Kentriki Makedonia | 2 | D, T | SA, ISA | The company needs technical consultancy for a digital platform that it has developed for conducting clinical studies and collecting real world data, fully customizable and scalable, easily adaptable to the different needs of its customers and different diseases, which will meet all international and national standards. The EDIH helps in the development of a prototype model (mockup), for a given protocol provided by the company. The company experiments with the inclusion of other protocols. | ongoing |
| 27 | private | micro-size | Peloponnisos | 2 | D, I, T | AI&DS | The company needed proof of concept and advice on a specific method it has developed that uses an algorithm to recognize neurological diseases from medical images. smartHEALTH checks the quality and precision, using two algorithmic models. As a conclusion, the method is a reliable diagnostic tool. The company also needs to develop cutting-edge algorithms for diagnosing neurological anomalies, with the use of medical imaging. smartHEALTH supports the development of a prototype for demonstrating the usage of medical images. The results of the testing are further processed for increasing accuracy, usability and operational issues. | ongoing |
| 28 | private | micro-size | Attica | 1 | - | - | - | - |
| 29 | private | micro-size | Attica | 2 | D, I, T | SA, HPC, BD | The company specializes in the field of genetics. It develops Polygenic Risk Scores (PRS) for various genetic traits and diseases, focusing on lifestyle and risk prediction. The EDIH offers consultation in order for the company to create a database management system to manage several processes, such as managing clients, collecting medical questionnaires and recording PRS results. The company receives a report with a description of the methodology followed by smartHEALTH experts thus the related interpretation of the results. Moreover, mockups of the UI system are delivered. | ongoing |
| 30 | private | small-size | Kentriki Makedonia | 1 | - | - | - | - |
| 31 | private | small-size | Attica | 1 | - | - | - | - |
| 32 | private | micro-size | Dytiki Elláda | 1 | - | - | - | - |
| 33 | private | small-size | Attica | 1 | - | - | - | - |
| 34 | private | micro-size | Kriti | 2 | I | AI&DS, SVPS, DT | Raw data preprocessing including data encoding and encryption, vital signs encoding and encryption and data denoising, techniques for abnormality detection and characterization with focus on ECG and auto labeling. | finalized |
| 35 | private | small-size | Kentriki Makedonia | 2 | D, T | SA, ISA | The company work for the development of an RPM (Remote Patient Monitoring) solution. It demonstrates its work, in regards to RPM dashboard development, to smartHEALTH experts, who in turn evaluate the approach and provide a literature review, for determining relevant state of the art work, including emerging technologies. Market research is also delivered to analyze the requested market. EDIH experts also develop a prototype model (mockup) and the directions concerning the design elements of the model that is developed. The company will test and experiment, using the model. | ongoing |
| 36 | private | micro-size | Kriti | - | - | - | - | - |
| 37 | private | micro-size | Attica | 1 | - | - | - | - |
| 38 | private | micro-size | Kentriki Makedonia | 1 | - | - | - | - |
| 39 | private | micro-size | Kriti | 2 | D, I | AI&DS, SVPS, DT | The company needs focuses on data anonymization, data imputation, data fusion, and trial reproducibility. | finalized |
| 40 | private | micro-size | Thessalia | 2 | D, I | AI&DS, SVPS, BD | The company needs services for data preprocessing including anonymization, denoising, imputation, augmentation and fusion. Additionally, the clients requested fostering integration services relevant to machine / deep learning techniques, including machine / deep learning algorithms, autoML and hyperparameter optimization, explainable AI and auto-labeling. | finalized |
| 41 | private | large-size | Attica | 2 | D, T | SA, ISA, CYB | The company intends to develop an EHR (Electronic Health Record) application and has no prior experience in this field. The company asked smartHEALTH experts to introduce the EHR concept to the company, including specialized technical consultation regarding interoperability, standards, privacy and cybersecurity. These, will help the company take the proper decisions, concerning the development of its application. The company also asked smartHEALTH experts to demonstrate the use of digital tools (APIs) for testing and experimenting with different interoperability standards. This testing & experimentation is expected to help substantially the company to develop their own application. | ongoing |
| 42 | private | small-size | Attica | 2 | D, T | SA, ISA, CLS, CYB | The company develops an EHR application. It needed to check that its application is compliant to the various requirements, for non-functional characteristics such as privacy, cybersecurity, interoperability, standards and regulations. | finalized |
| 43 | private | micro-size | Attica | 2 | D | SA, CLS, CYB | The company was in need to assess its IT platform. The company had developed it without being completely aware of the formal requirements, applicable to medical software. At the same time, the company needed guidance on how to further develop the platform for hosting a much higher number of users. The smartHEALTH experts reviewed the platform and additionally, they provided technical consultation for its expansion. | finalized |
| 44 | private | micro-size | Kriti | 2 | D, T | AI&DS, IBT, SE | The company has the intention to invest in the creation of an advanced tool, using AI, regarding medical imaging and diagnosis of diseases. EDIH experts present current & future AI solutions to the company. This, along with the proper technical consultation will help the company to select the most suitable method for developing a competitive solution. The company also works for developing an innovative drug for Parkinson's and other neurodegeneration diseases. The smartHEALTH EDIH will provide experimental design guidance to evaluate the effectiveness and safety of the chemical molecules it develops in clinical trials. This testing process will incorporate in-silico (digital) modeling of the binding, aiming at the physicochemical properties of the molecules and whether they simulate the desired drug properties. Safety of the molecule will be assessed whether the different structures raise issues of unacceptable side effects in clinical trials, combining experimental data with algorithms that correlate structures with potential toxicity issues. | ongoing |
| 45 | public | large-size | Kriti | - | - | - | - | - |
| 46 | public | large-size | Attica | - | - | - | - | - |
| 47 | public | large-size | Kentriki Makedonia | 2 | F | SA, ISA | The public hospital has taken the initiative for creating a National Arthroplasty Register. Greece lacks such a registry. The provision of consulting services from smartHEALTH support the hospital in the creation of the first national register of arthroplasty in Greece, which aims to record and monitor the progress of hip and knee arthroplasties at a national level. The ultimate goal of the project is to optimize the provided medical care and the finances of Health, through its digitalization. The registry that will be created will be open to all the relevant doctors/authorities in the country. | ongoing |
| 48 | public | large-size | Attica | 1 | - | - | - | - |
| 49 | public | large-size | Kentriki Makedonia | 2 | D, I, T | AI&DS | The public hospital, as part of the EHDEN community, has transformed its database according to the OMOP Common Data Model (CDM). Experts from smartHEALTH perform data quality assessment. Additionally, an AI-supported platform is designed, tested, and prototyped to support pharmacovigilance queries using the OMOP-CDM format. This initiative enables the Hospital to lead at the national level in the secondary use of real-world data. | ongoing |
| 50 | public | large-size | Attica | 2 | D, I, T | AI&DS, CNLP | The hospital focuses on the prevention and treatment of cardiovascular diseases. It provides high-quality, patient-centric healthcare that is adapted to the new knowledge that comes from its research activity. In addition, it stands out for its strategy in the sector of innovation, as it promotes the adoption of innovative technologies and methods. EDIH experts offer consultation services in the field of Clinical NLP and AI. The aim is that existing unstructured, non-digital clinical data are homogenized and collected together into appropriate data structures for efficient data analysis. Using clinical NLP technologies, a prototype clinical decision support system is created. | ongoing |
| 51 | public | large-size | Kriti | - | - | - | - | - |

TABLE S1. The data set includes a list of applications formally submitted. Information includes type of organization (private vs. public), staff size (micro-size: 1-9 staff, small-size: 10-49 staff, medium-size: 50-249 staff, and large-size: +250 staff), region (based on basic regions classification for regional policies (NUTS2), as defined by Eurostat – European Commission), and current phase (1 or 2 for the ones contracted that focus on test-before-invest, - for the rest). For those that have entered phase 2 for test-before-invest services, it includes type of service (D for demonstration and proof-of-concept, I for fostering integration, T for testing, prototyping & experimentation, and F for flagship), technology used (SA for software architectures, AI&DS for artificial intelligence & decision support, CPS for cyber physical systems, ISA for internet services & applications, CLS for cloud services, SVPS for sensors & vision processing systems, CYB for cybersecurity, HPC for high-performance computing, BD for big data, IBT for industrial biotechnology, DT for digital twins, SE for simulation engineering, and CNLP for clinical natural language processing), short description, and status (ongoing or finalized)
